# Eugenol triggers apoptosis in breast cancer cells through E2F1/survivin down-regulation

**DOI:** 10.1186/1471-2407-13-600

**Published:** 2013-12-13

**Authors:** Ibtehaj Al-Sharif, Adnane Remmal, Abdelilah Aboussekhra

**Affiliations:** 1 Department of Molecular Oncology, King Faisal Specialist Hospital and Research Center, MBC # 03-66, PO BOX 3354, Riyadh 11211, Saudi Arabia; 2 Faculté des Sciences Fès, Laboratoire de Biotechnologie Atlas, Fès, Morocco

**Keywords:** Apoptosis, Breast cancer, Eugenol, E2F1, Survivin

## Abstract

**Background:**

Breast cancer is a major health problem that threatens the lives of millions of women worldwide each year. Most of the chemotherapeutic agents that are currently used to treat this complex disease are highly toxic with long-term side effects. Therefore, novel generation of anti-cancer drugs with higher efficiency and specificity are urgently needed.

**Methods:**

Breast cancer cell lines were treated with eugenol and cytotoxicity was measured using the WST-1 reagent, while propidium iodide/annexinV associated with flow cytometry was utilized in order to determine the induced cell death pathway. The effect of eugenol on apoptotic and pro-carcinogenic proteins, both *in vitro* and in tumor xenografts was assessed by immunoblotting. While RT-PCR was used to determine eugenol effect on the E2F1 and survivin mRNA levels. In addition, we tested the effect of eugenol on cell proliferation using the real-time cell electronic sensing system.

**Results:**

Eugenol at low dose (2 μM) has specific toxicity against different breast cancer cells. This killing effect was mediated mainly through inducing the internal apoptotic pathway and strong down-regulation of E2F1 and its downstream antiapoptosis target survivin, independently of the status of p53 and ERα. Eugenol inhibited also several other breast cancer related oncogenes, such as NF-κB and cyclin D1. Moreover, eugenol up-regulated the versatile cyclin-dependent kinase inhibitor p21^WAF1^ protein, and inhibited the proliferation of breast cancer cells in a p53-independent manner. Importantly, these anti-proliferative and pro-apoptotic effects were also observed *in vivo* in xenografted human breast tumors.

**Conclusion:**

Eugenol exhibits anti-breast cancer properties both *in vitro* and *in vivo*, indicating that it could be used to consolidate the adjuvant treatment of breast cancer through targeting the E2F1/survivin pathway, especially for the less responsive triple-negative subtype of the disease.

## Background

Breast cancer remains a worldwide public health concern and a major cause of morbidity and mortality among females [1]. Treatment of breast cancer includes, tumor resection, radiation, endocrine therapy, cytotoxic chemotherapy and antibody-based therapy [2]. However, resistance to these forms of therapies and tumor recurrence are very frequent. Furthermore, there is relative lack of effective therapies for advanced-stage and some forms of the disease such as triple negative breast cancer (TNBC). Recently, PARP inhibitors showed promising results against tumors with mutated BRCA1 and TNBC [3,4]. Therefore, scientists keep seeking for new agents with higher efficiency and less side effects. Of 121 prescription drugs in use for cancer treatment, 90 are derived from plant species and 74% of these drugs were discovered by investigating a folklore claim [5,6]. Indeed, several natural products and dietary constitutes exhibit anti-cancer properties without considerable adverse effects [7,8]. Therefore, the abundance of flavonoids and related polyphenols in the plant kingdom makes it possible that several hitherto uncharacterized agents with chemopreventive or chemotherapeutic effects are still to be identified. Several of these products such as curcumin, green tea, soy and red clover are currently in clinical trials for the treatment of various forms of cancer [9].

Eugenol (4-allyl (-2-mthoxyphenol)), a phenolic natural compound available in honey and in the essential oils of different spices such as *Syzgium aromaticum* (clove), *Pimenta racemosa* (bay leaves), and *Cinnamomum verum* (cinnamon leaf), has been exploited for various medicinal applications. It serves as a weak anaesthetic and has been used by dentists as a pain reliever and cavity filling cement (“clove oil”). In Asian countries, eugenol has been used as antiseptic, analgesic and antibacterial agent [10]. In addition, eugenol has antiviral [11], antioxidant [12] and anti-inflamatory functions. Furthermore, while it has been proved not to be carcinogenic neither mutagenic [13], eugenol has several anti-cancer properties. Indeed, eugenol has antiproliferative effects in diverse cancer cell lines as well as in B16 melanoma xenograft model [14-16]. Eugenol induced apoptosis in various cancer cells, including mast cells [17], melanoma cells [15] and HL-60 leukemia cells [18]. Moreover, eugenol induced apoptosis and inhibited invasion and angiogenesis in a rat model of gastric carcinogenesis induced by MNNG [19]. Interestingly, Eugenol is listed by the Food and Drug Administration (FDA) as “Generally Regarded as Safe” when consumed orally, in unburned form.

In the present paper we present clear evidence that eugenol has potent anti-breast cancer properties both *in vitro* and *in vivo* with strong inhibitory effect on E2F1 and survivin.

## Methods

### Ethics statement

Animal experiments were approved by the KFSH & RC institutional Animal Care and Use Committee (ACUC) and were conducted according to relevant national and international guidelines**.** Animals suffered only minimal pain due to needle injection and certain degree of distress related to the growth/burden of the tumor. Euthanasia was performed using CO2 chamber.

### Cell lines, chemicals and cell culture

All cell lines were purchased from the American Type Culture Collection (ATCC) and cultured according to ATCC instructions. The p53 and ER-α status of these cells are mentioned in Table 1. MCF7, T47-D and MDA-MB-231 were maintained in RPMI-1640 (Gibco, Grand Island, NY, USA), L-glutamine 1%, 10% fetal bovine serum (FBS), 1% antibiotic/anti-mycotic (penicillin/streptomycin) (Sigma Aldrich, St Louis, MO, USA). MCF 10A cells were cultured in universal medium: (1:1 mixture of Dulbecco’s Modified Eagles Medium (DMEM) and Ham’s F12 medium (Gibco) supplemented with 5% FBS, 1% antibiotic antimycotic, 20 ng/ml epidermal growth factor (EGF), 100 ng/ml choleratoxin, 10 μg/ml insulin, and 500 ng/ml hydrocortisone). Cells were maintained at 37°C in humidified incubator with 5% CO_2_. Eugenol (Sigma) was diluted in DMSO and prepared at 1 mM.

**Table 1:** Features of used cell lines

**Cell lines**	**p53 status**	**ER-α**	**LC**_**50 **_**(μM)**
MDA-MB-231	mutant	negative	1.7
MCF7	wild-type	positive	1.5
T47-D	wild-type	positive	0.9
MCF 10A	wild-type	positive	2.2

### Cytotoxicity assay

Cells were seeded into 96-well plates at 0.5-1.10^4^/well and incubated overnight. The medium was replaced with fresh one containing the desired concentrations of eugenol. After 20 hrs, 10 μl of the WST-1 reagent (Roche Diagnostics, Mannheim, Germany) was added to each well and the plates were incubated for 4 hrs at 37°C. The amount of formazan was quantified using ELISA reader at 450 nm of absorbance.

### Cell proliferation analysis

Complete medium (100 μl) containing 2–4 x 10^3^ cells was loaded in each well of the 96-well microtiter E-plates with integrated microelectronic sensor arrays at the bottom of each well. The plate was incubated for at least 30 min in a humidified, 37°C, 5% CO2 incubator, and then was inserted into the Real-Time Cell Electronic Sensing System (RT-CES system, xCELLigence system from Roche Applied Science, originally invented by the US company ACEA Biosciences Inc., San Diego, CA). This allows for label-free and dynamic monitoring of cell proliferation. Cells were monitored for 90 hrs. The electronic readout, cell-sensor impedance is displayed as arbitrary units called cell index, which is defined as Rn-Rb/Rb, with Rn = cell-electrode impedance of the well with the cells and Rb = the background impedance of the well with the media alone.

### Cellular lysate preparation

Cells were washed with PBS and then scraped in RIPA buffer (150 mM NaCl, 1 mM EDTA, 1% Nonidet P-40, 0.5% Sodium deoxycolate, 0.1% SDS, 50 mM Tris–HCl (pH 7.5)), supplemented with protease inhibitors. Lysates were homogenized and then centrifuged at 14000 r.p.m at 4°C for 15 min in an eppendorf micro centrifuge. The supernatant was removed, aliquoted and stored at -80°C.

### Immunoblotting

SDS-PAGE was performed using 12% separating minigels and equal amount of proteins were loaded. After protein migration and transfer onto polyvinylidene difluroide membrane (PVDF), the membrane was incubated overnight with the appropriate antibodies:

E2F1 (KH95), Survivin (D-8), NF-κB (F-6), p21 (F-5), Bax (B-9), Bcl-2 (C-2), Cyclin D1 (HD11), caspase-9 (F-7), Cox-2 (29), and β-Catenin (9 F2) were purchased from Santa Cruz, Biotechnology (Santa Cruz, CA, USA); Cleaved caspase-3 (Asp175), Cleaved caspase-9 (Asp 315), Cleaved-PARP-1 (ASP 214), Cytochrome C and GAPDH were purchased from Cell Signaling (Danvers, MA, USA).

Visualization of the second antibody was performed using the superSignal West Pico Chemiluminescent substrate according to the manufacturer’s recommendations (THERMO Scientific, Rockford, IL).

### RNA extraction, cDNA synthesis and RT-PCR

Total RNA was extracted using the Tri® Reagent (Sigma) and the yield was quantitated spectrophotometrically. Following the manufacturer’s instructions, single stranded cDNA was synthesized using 200 ng of total RNA, the MMLV Reverse Transcriptase and the oligo dT_18_ (Roche, San Francisco, CA, USA). The cDNA was amplified for 40 cycles under the following conditions: melting temperature (95°C) for 50 seconds, annealing temperature (54°C) for 50 seconds, and extension temperature (72°C) for 1 min. The RT-PCR products were separated by electrophoresis on a 2% agarose gel at 80 V for an hour. The sequences of the primers were as follow:

***β-actin***, Fw:5′- CCCAGCACAATGAAGATCAAGATCAT; Rv: 5′-ATCTGCTGGAAGGTGGACAGCGA.

**S** ***urvivin***, Fw: 5′- CAGAGGAGGCGCCAAGACAG; Rv: 5′-CCTGACGGCGGAAAACGC.

***E2F1***, Fw: 5′-ATGTTTTCCTGTGCCCTGAG; Rv: 5′-ATCTGTGGTGAGGGATGAGG.

### Quantification of protein and RNA expression levels

The expression levels of RNAs and proteins were measured using the densitometer (BIO-RAD GS-800 Calibrated Densitometer, USA). Films were scanned and protein signal intensity of each band was determined. Next, dividing the obtained value of each band by the values of the corresponding internal control allowed the correction of the loading differences. The fold of induction was determined by dividing the corrected values that corresponded to the treated samples by that of the non-treated one (time 0).

### Annexin V/PI and flow cytometry

Cells were treated either with DMSO or eugenol, and then were reincubated in complete media. Detached and adherent cells were harvested 72 hrs later, centrifuged and re-suspended in 1 ml PBS. Cells were then stained by PI and Alexa Fluor 488 annexinV, using Vibrant Apoptosis Assay kit #2 (Molecular probe, Grand Island, NY, USA). Stained cells were analyzed by flow cytometry. The percentage of cells was determined by the FACScalibur apparatus and the Cell Quest Pro software from Becton Dickinson, USA. For each cell line 3 independent experiments were performed.

### shRNA transfection

The transfection using *E2F1*-shRNA and control–shRNA was performed using Lipofectamine (Life technologies, Grand Island, NY, USA) as previously described [20].

### Tumor xenografts

Breast cancer xenografts were created in 10 nude mice by subcutaneous injection of the MDA-MB-231 cells (5.10^6^) into the right leg of each mouse. After the growth of the tumors (about 2 cm^3^) the animals were randomized into 2 groups to receive intraperitoneal (i.p.) injections of eugenol (100 mg/kg) or the same volume of DMSO each 2 days for 4 weeks. Tumor size was measured with a calliper using the following formula (Length X Width X Height).

## Results

### Eugenol has cytotoxic effect on estrogen positive and negative breast cancer cells

**Figure 1:**
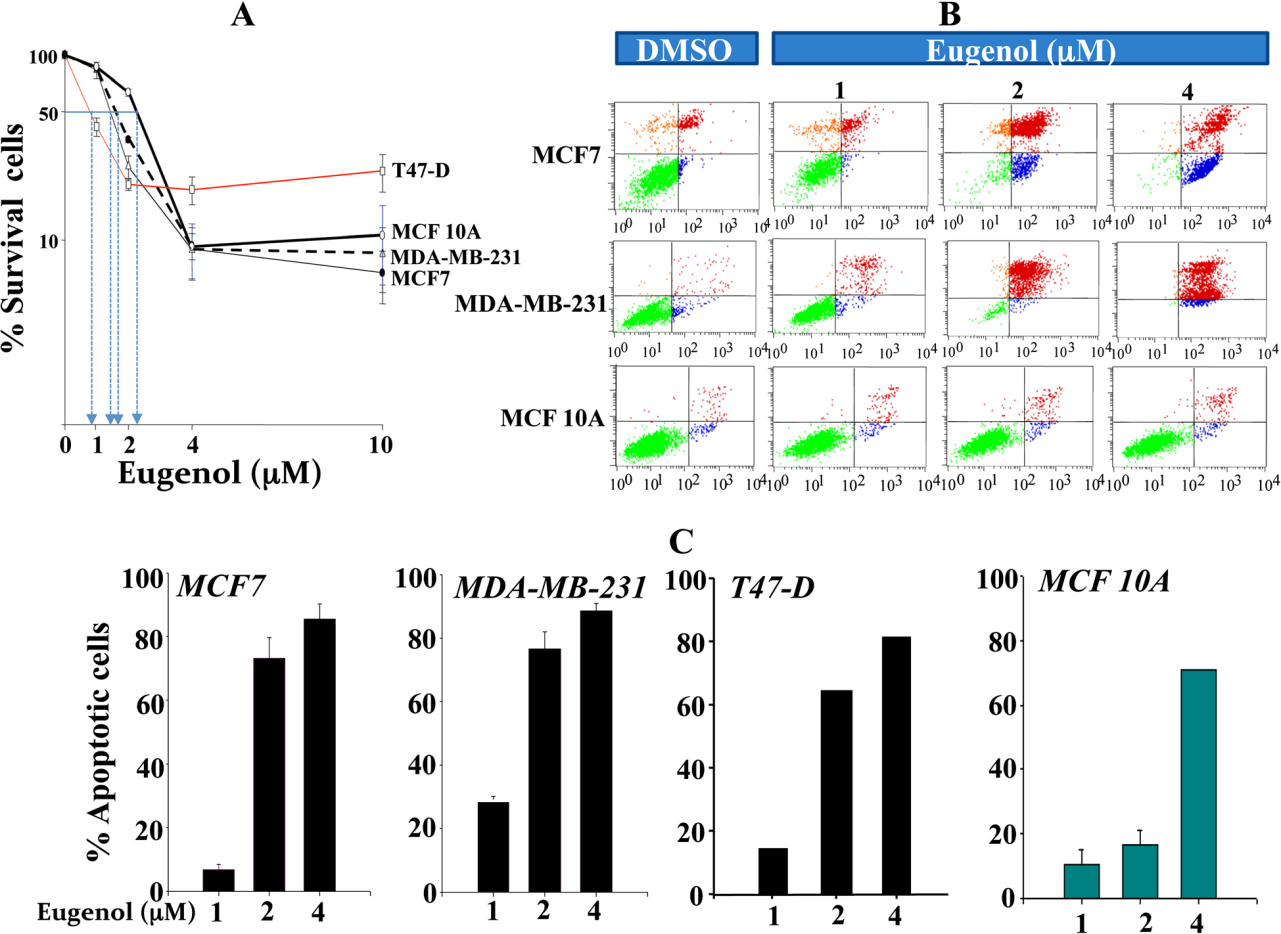
**Cytotoxic effects of eugenol on breast cancer cells. (A)** Exponentially growing cells were cultured in 96 well plates and treated with the indicated concentrations of eugenol for 24 hrs. Cell death was analyzed using the WST-1 assay. The arrows indicate the LC_50_ for each cell line. Error bars represent means ± S.D. **(B)** Cells were either sham-treated (DMSO) or challenged with the indicated concentrations of eugenol for 72 hrs, and then cell death was assessed by PI/annexinV/flow cytometery. **(C)** Histograms presenting the proportions of induced apoptosis in the various cell lines. Data are presented as means ± S.D.

We first investigated the cytotoxic effect of eugenol on different breast cancer cells (MDA-MB-231, MCF7 and T47-D) and the non-tumorigenic MCF 10A cell line using the WST-1 assay. Cells were seeded in triplicates into microtiter plates and treated with increasing concentrations of eugenol for 24 hrs, and then the cytotoxic effect was measured. While MCF 10A cells exhibited high resistance to eugenol, with an LC_50_ (the concentration that leads to 50% survival) of 2.4 μM, breast cancer cells showed clear sensitivity (Figure 1A). The LC_50_ were 1.7 μM, 1.5 μM and 0.9 μM for MDA-MB-231, MCF7 and T47-D, respectively (Figure 1A, Table 1). This indicates that eugenol has differential cytotoxicity against different breast cancer cell lines, but its less toxic against non-neoplastic breast epithelial cells.

### Eugenol triggers apoptosis in breast cancer cells through the mitochondrial pathway independently of the estrogen receptor status

Next, we investigated whether eugenol triggers apoptosis in breast cancer cells. To this end, cells were treated with different concentrations of eugenol for 3 days, and then were stained with annexin V/Propidium Iodide (PI), and were sorted by flow cytometry. Figure 1B shows that eugenol triggered essentially apoptosis in both breast cancer cells MCF7 and MDA-MB-231. However, the non-carcinogenic MCF 10A cells exhibited great resistance. Figure 1C shows the proportions of eugenol-induced apoptosis, which was considered as the sum of both early and late apoptosis after deduction of the proportion of spontaneous apoptosis. Interestingly, eugenol effect increased in a dose-dependent manner in the 4 cell lines (Figure 1C). While the effect was only marginal in response to 1 μM, the proportion of apoptotic cells reached 80% in MCF7 and MDA-MB-231 and 65% in T47-D, while it was only 20% in MCF 10A in response to 2 μM eugenol. At 4 μM, eugenol was toxic for MCF 10A as well, and apoptosis reached 70% in these cells, while it was beyond 80% in the three breast cancer cell lines (Figure 1C). This indicates that the eugenol-dependent cytotoxicity is mediated mainly through the apoptotic cell death pathway, with selective effect on breast cancer cells up to 2 μM. Therefore, this concentration was used for the next experiments.

**Figure 2:**
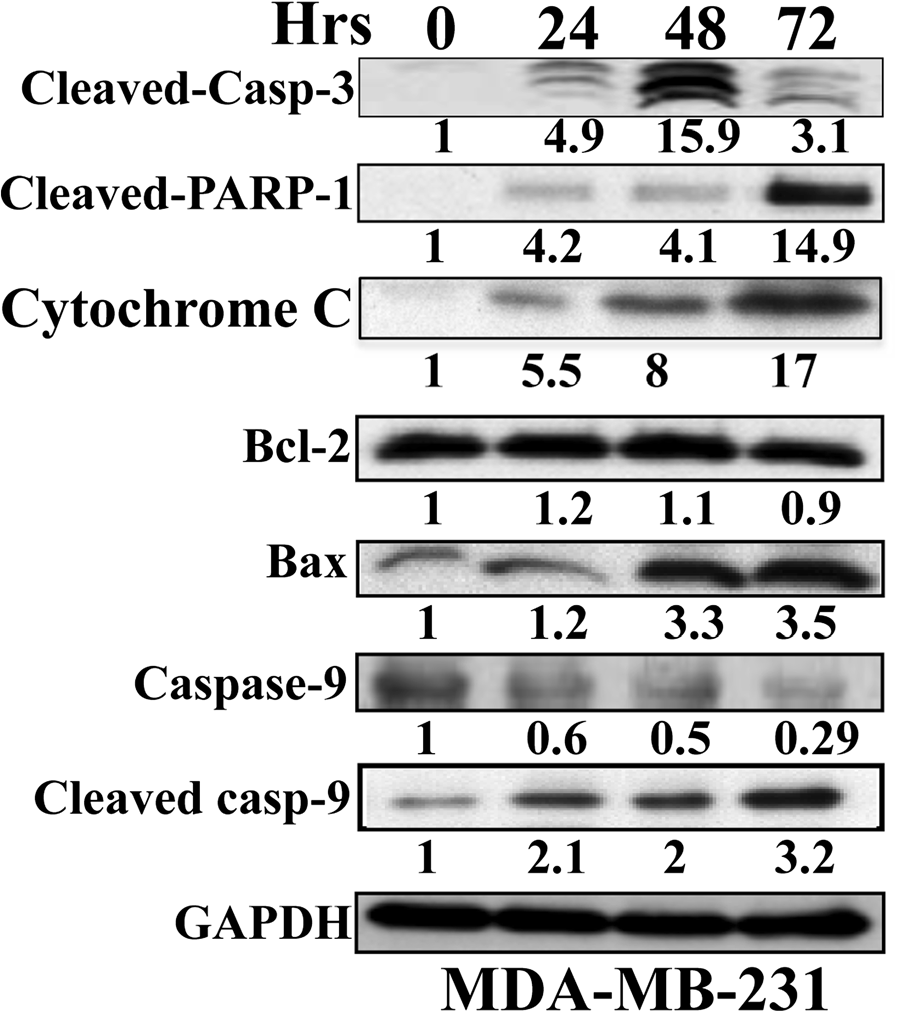
**Eugenol triggers apoptosis through the mitochondrial pathway.** MDA-MB-231 cells were treated with eugenol (2 μM), and then were harvested at the indicated periods of time. Proteins (50 μg) were used for western blot analysis utilizing antibodies against the indicated proteins. The numbers below the bands represent the corresponding expression levels as compared with time 0 and after normalization against GAPDH.

To confirm the induction of apoptosis by eugenol in breast cancer cells and determine the apoptotic route that eugenol activates, MDA-MB-231 cells were treated with eugenol (2 μM) and were harvested after different time periods (0, 24, 48 and 72 hrs). Whole cell extracts were prepared and were used to evaluate the levels of different pro- and anti-apoptotic proteins using the immunoblotting technique and specific antibodies. GAPDH was used as internal control. First, we assessed the effect of eugenol on the caspase-3 and PARP-1 proteins (two principal markers of apoptosis). Figure 2 shows that eugenol triggered the cleavage of caspase-3 and PARP-1, which led to significant increase in their active forms, confirming the induction of apoptosis by eugenol in breast cancer cells. Next, we assessed the effect of eugenol on the levels of Bax and Bcl-2 and have found that while the level of Bax increased in a time-dependent manner, the level of Bcl-2 did not change (Figure 2). This resulted in a time-dependent increase in the Bax/Bcl-2 ratio reaching a level 4 fold higher after 72 hrs of treatment, suggesting that eugenol triggers apoptosis through the mitochondrial pathway. To confirm this, we assessed the levels of cytochrome C, caspase 9 and its active form in these cells, and showed that while the level of caspase-9 decreased in a time-dependent manner reaching a level more than 3 fold lower after 72 hrs of treatment, the level of cleaved caspase-9 and cytochrome C increased 3 fold, and 17 fold, respectively (Figure 2). Together, these results demonstrate that eugenol triggers apoptosis in breast cancer cells through the internal mitochondrial pathway via Bax increase.

### Eugenol is an efficient inhibitor of several cancer promoting genes

**Figure 3:**
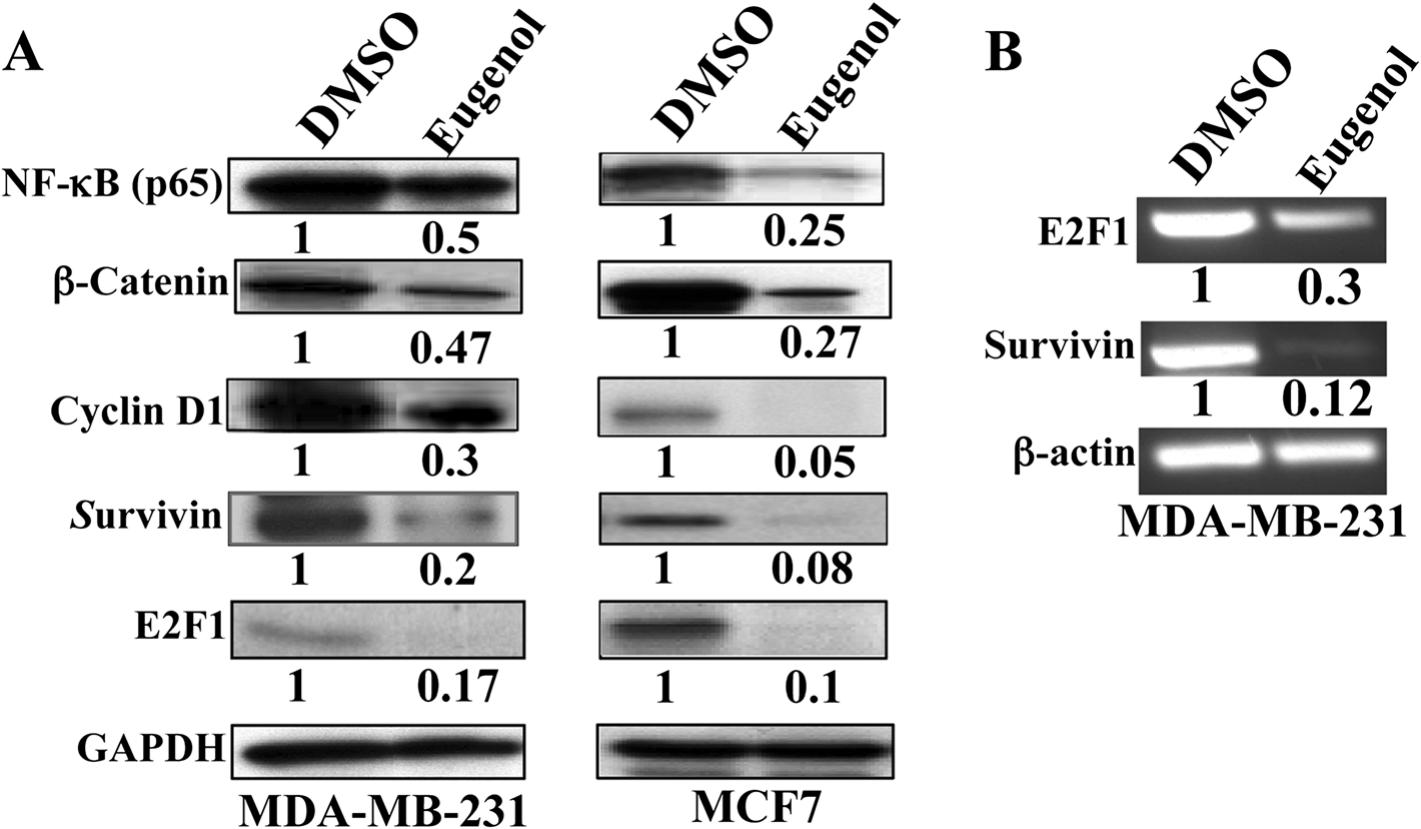
**Eugenol suppresses the expression of several oncoproteins. (A)** Cells were either sham-treated (DMSO) or challenged with eugenol (2 μM) for 24 hrs. Subsequently, cells were harvested and proteins were used for western blot analysis using the indicated antibodies. The numbers under the bands represent the corresponding expression levels as compared to time 0 and after normalization against GAPDH. **(B)** DMSO- and eugenol-treated cells (2 μM) were harvested after 4 hrs, and total RNA was extracted and subjected to RT-PCR using specific primers for the indicated genes. The resulting products were electrophorezed in ethidium bromide stained 2% agarose gel. The numbers under the bands represent the corresponding expression levels as compared to control (DMSO) and after normalization against β-actin.

To investigate the effect of eugenol on cancer-related genes, MDA-MB-231 and MCF7 cells were either sham-treated (DMSO) or challenged with eugenol (2 μM) for 24 hrs, and then cell lysates were prepared and protein levels were monitored by immunoblotting. Eugenol-treatment had strong effect on the expression of NF-κB, decreasing its level 2 fold and 3 fold in MDA-MB-231 and MCF7, respectively (Figure 3A). Similar effect was observed on β-catenin, indicating that eugenol could inhibit both major cancer promoting pathways Akt/NF-κB and Wnt/β-catenin. To confirm this, we studied the effect of eugenol on the common downstream effector cyclin D1 [21-23]. Indeed, eugenol treatment decreased cyclin D1 level 3 fold in MDA-MB-231 cells and 20 fold in MCF7 cells (Figure 3A). Interestingly, the strongest eugenol inhibitory effect was observed on E2F1 and survivin, a cancer anti-apoptosis marker [24] in both cell lines (Figure 3A). Indeed, after 24 hrs of treatment, the E2F1 and survivin proteins became almost undetectable (Figure 3A). To ascertain the level of action of eugenol on these genes, we investigated the effect on their mRNA levels. To this end, MDA-MB-231 cells were treated with eugenol (2 μM) for 24 hrs and total RNA was purified and amplified using RT-PCR and specific primers. Interestingly, eugenol treatment reduced the expression level of both transcripts (Figure 3B). This indicates that eugenol inhibits the expression of these 2 genes at the transcriptional or post-transcriptional level. Therefore, eugenol targets several breast cancer-related signaling pathways, leading to strong inhibition of two important breast cancer oncogenes E2F1 and survivin in both luminal as well as basal like breast cancer cell lines.

### Eugenol triggers apoptosis through E2F1/survivin down-regulation

**Figure 4:**
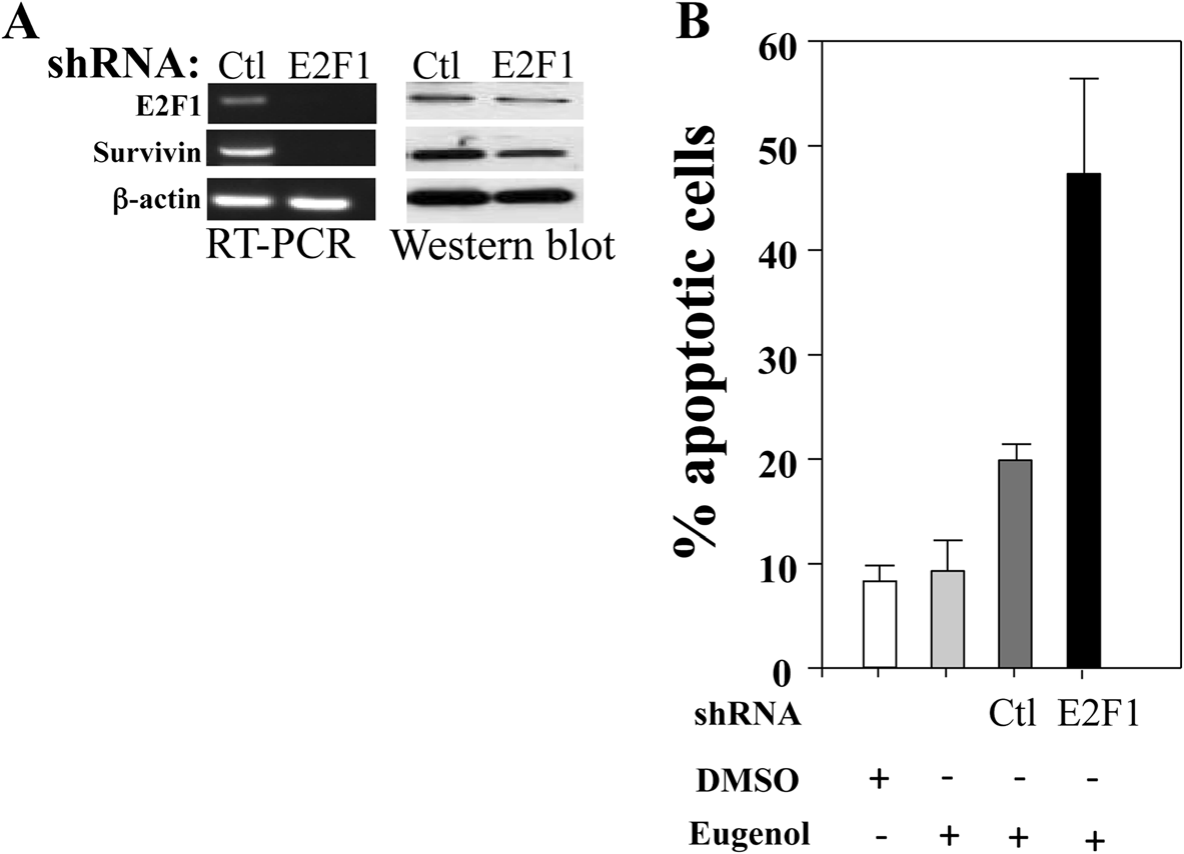
**Eugenol-dependent apoptosis is mediated through down-regulation of E2F1 and survivin. (A)** Total RNA and proteins were extracted from MDA-MB-231 cells expressing either control-shRNA or E2F1-shRNA and used for RT-PCR and western blot analysis. **(B)** MDA-MB-231 cells were treated as shown for 72 hrs, and then apoptosis was assessed by annexinV/PI like in Figure 1B. Data are presented as means ± S.D.

To elucidate the role of eugenol-related down-regulation of E2F1 and its antiapoptosis target survivin [25] in apoptosis induction in breast cancer cells, we studied the effect of E2F1 specific down-regulation on the cytotoxic effect of eugenol. Therefore, MDA-MB-231 cells were transiently transfected with specific E2F1-shRNA or control-shRNA. Figure 4A shows the effect of E2F1-shRNA on the level of the E2F1 mRNA and protein. Interestingly, like eugenol, E2F1 down-regulation by specific shRNA reduced also the expression level of the survivin mRNA and protein (Figure 4A). This shows that E2F1 controls the expression of survivin in these cells. We next treated MDA-MB-231 cells expressing either control-shRNA or E2F1-shRNA with DMSO or eugenol (1 μM) for 48 hrs. Figure 4B shows that 1 μM eugenol had only marginal effect on MDA-MB-231 cells. Interestingly, E2F1 down-regulation doubled the killing effect of eugenol as compared to the effect on the corresponding control cells (Figure 4B). This suggests that the killing effect of eugenol is mediated through E2F1/survivin down-regulation.

### Eugenol inhibits cell proliferation and up-regulates p21^WAF1^ in breast cancer cells

**Figure 5:**
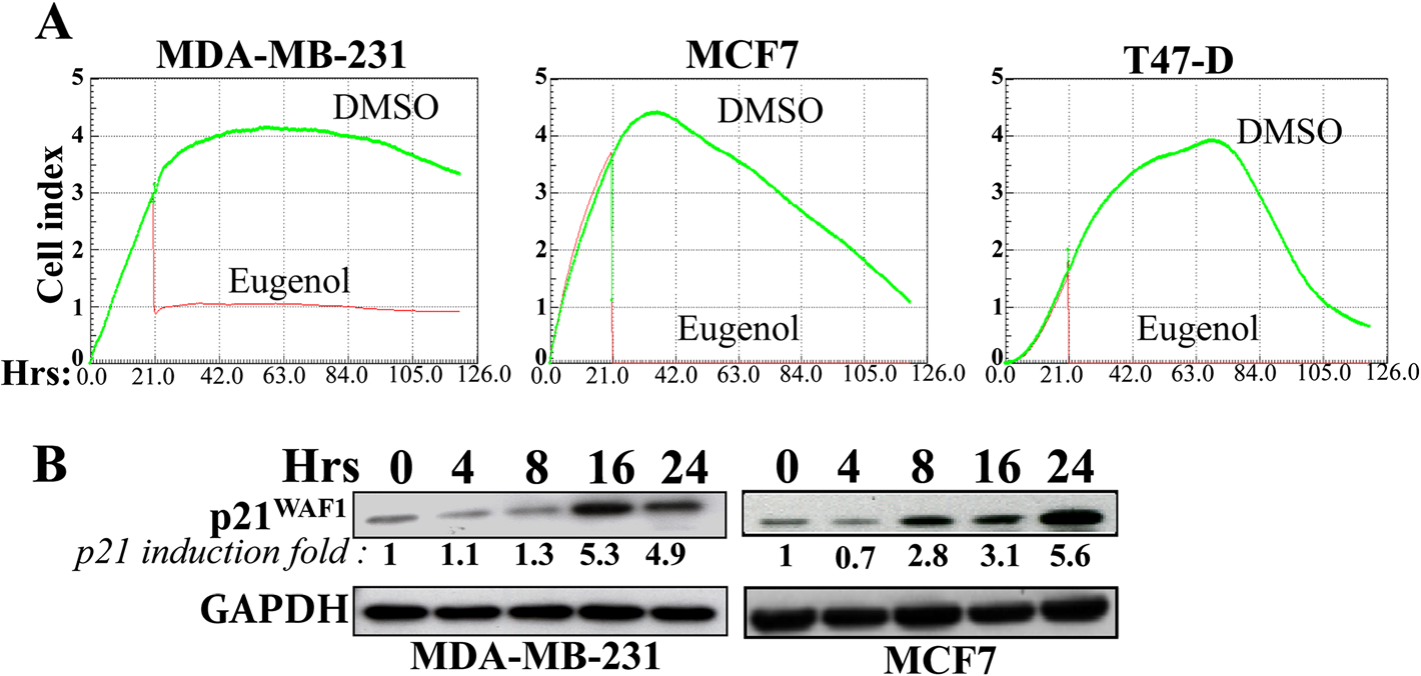
**Eugenol inhibits breast cancer cell proliferation and up-regulates p21**^**WAF1**^**. (A)** Sub-confluent cells (2–4.10^3^) were either sham-treated or challenged with eugenol (2 μM) for the indicated periods of time, and cell proliferation rate was determined using the Real-Time Cell Electronic Sensing System. **(B)** MDA-MB-231 and MCF7 cells were treated with eugenol (2 μM) for the indicated periods of time, and then cell lysates were prepared and 50 μg of proteins were used for western blot analysis utilizing the indicated antibodies.

Exponentially growing breast cancer cells (MDA-MB-231, MCF7 and T47-D) were seeded in 96-well plates and were either sham-treated with DMSO or challenged with eugenol (2 μM), and then reincubated for 120 hrs. During this time, the real-time cell electronic sensing system was used to monitor cell proliferation. While DMSO-treated cells continued to proliferate, eugenol treatment suppressed cell proliferation in the 3 breast cancer cell lines (Figure 5A).

Next, we evaluated the effect of eugenol on the expression of the versatile cyclin-dependent kinase inhibitor p21^WAF1^ in MDA-MB-231 and MCF7. After treatment with eugenol (2 μM) cells were harvested at different periods of time (0–24 hrs) and immunoblotting was utilized for protein level assessment using specific antibodies. Figure 5B shows that eugenol increased the level of p21^WAF1^ reaching a level 5 fold higher as compared to the basal level in both cell lines. Therefore, eugenol is a strong inducer of p21^WAF1^ expression in a p53-independent manner.

### Eugenol inhibits tumor growth of breast tumor xenografts in mice

**Figure 6:**
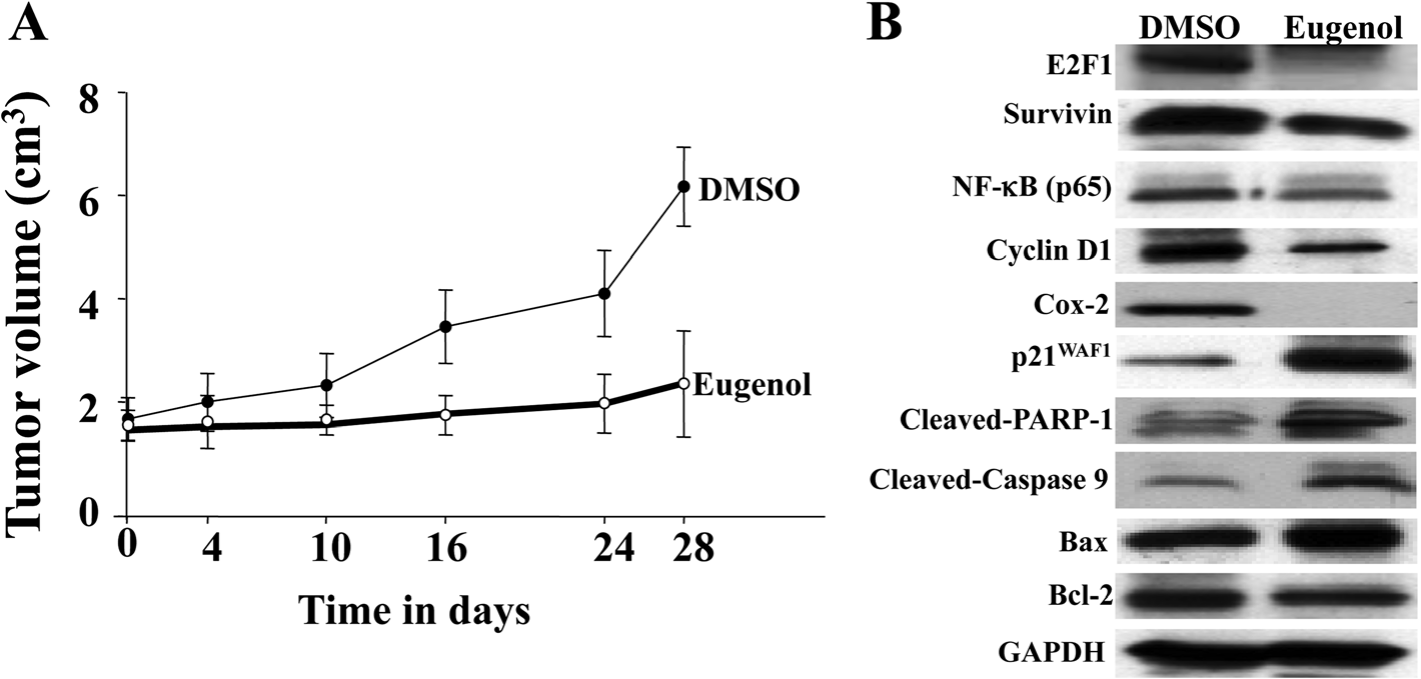
**Eugenol inhibits tumor growth and modulates gene expression *in vivo.*** Breast cancer xenografts were created by injecting MDA-MB-231 cells subcutaneously into nude mice. When tumors grew, eugenol was given i.p. at a dose of 100 mg/kg. Control animals were treated with DMSO. **(A)** Tumor growth. Data are presented as means ± S.D. **(B)** Following the treatments, tumors were excised and protein extracts were prepared and used for immunoblotting analysis using the indicated antibodies.

To study the anti-cancer effect of eugenol *in vivo*, breast cancer xenografts were created by injecting 5.10^6^ MDA-MB-231 cells subcutaneously into nude mice. When tumors reached a reasonable volume (about 2 cm^3^), eugenol was given i.p. at a dose of 100 mg/kg each 2 days for 4 weeks. Control animals were treated with DMSO only. Interestingly, in the mock-treated animals, the volume of the tumors increased in a time-dependent manner and became 3 fold bigger than the initial ones (Figure 6A). On the other hand, treatment with eugenol inhibited tumor growth (Figure 6A). This shows that eugenol inhibits the proliferation of breast cancer cells *in vivo* as well.

Subsequently, we investigated the effect of eugenol on the expression of various cancer-related genes in tumor xenografts. Figure 6B shows that eugenol down-regulated E2F1 and survivin in tumor xenografts as well. Concomitantly, the levels of NF-κB and cyclin D1 also decreased and Cox-2 became undetectable (Figure 6B). Interestingly, like *in vitro*, eugenol up-regulated p21^WAF1^ (Figure 6B). Furthermore, we have investigated the effect of eugenol on the expression of apoptosis-related genes and have shown that eugenol increased the levels of Bax, cleaved PARP-1 and the active form of caspase-9, but decreased the level of the anti-apoptosis protein Bcl-2, suggesting eugenol-dependent induction of apoptosis *in vivo* and confirming the results obtained *in vitro* (Figure 6B).

## Discussion

In the present study we have shown that eugenol, a natural phenolic compound, exhibits strong anti-breast cancer features. Indeed, we present here clear evidence that eugenol could be considered as a potential therapeutic agent for both ER-negative as well as ER-positive breast tumors for the following reasons:

First, eugenol is cytotoxic and triggered apoptosis in great proportion of breast cancer cells, with marginal effect on normal cells in response to 2 μM of eugenol. However, at higher concentration (4 μM), eugenol killed normal cells as well, showing that this molecule may have some toxicity when used as high concentrations.

Eugenol-related apoptosis was mediated through the mitochondrial pathway via Bax increase, and is p53- and ERα-independent since it occurred in p53- and ERα-defective cells, MDA-MB-231 [26]. This effect was mediated through strong down-regulation of E2F1 and its antiapoptosis target survivin [25]. Indeed, specific down-regulation of E2F1 strongly reduced the level of survivin and increased the effect of eugenol on breast cancer cells (Figure 4). Notably, low E2F1 levels were related to favorable breast cancer outcome [27]. On the other hand, E2F1 expression was related with poor survival of lymph node-positive breast cancer patients treated with fluorouracil, doxorubicin and cyclophosphamide [28]. This indicates that high E2F1 levels reduce the response of breast tumors to therapy. Similarly, while survivin expression has been found to confer resistance to chemotherapy and radiation, targeting survivin in experimental models improved survival [29]. Thereby, the fact that eugenol can inhibit both E2F1 and survivin *in vitro* and in tumor xenografts, indicates that eugenol could be used to consolidate the adjuvant treatment of breast cancer patients, especially the clinically aggressive ER-negative types, whose prognosis is still poor and clinically characterized as more aggressive and less responsive to standard treatments [30,31].

Second, eugenol is a potent inhibitor of cell proliferation, may be through inhibition of E2F1 and great increase in the level of the cyclin-dependent kinase inhibitor p21^WAF1^*in vitro* and in tumor xenografts. E2F1 is a transcription factor that regulates the expression of several genes involved in G1 to S phase transition [32]. In a previous study it has been shown that eugenol inhibits cell proliferation in melanoma cells through inhibition of E2F1 [15]. p21 induction in p53-defective MDA-MB-231 cells, suggests the ability of eugenol to induce p21^WAF1^ through p53-independent mechanism. Overexpression of p21^WAF1^ can block both the G1/S and G2/M transitions of the cell cycle [33]. Furthermore, p21^WAF1^ is a modulator of apoptosis in a number of systems [34-36]. Therefore, the strong eugenol-dependent up-regulation of p21^WAF1^ in a p53-independent manner could be of great value for the inhibition of cancer cell proliferation and the induction of cell death in various p53-defective breast tumors, including the triple negative form of the disease where p53 deficiency is observed in up to 44% [37].

Third, eugenol down-regulated several onco-proteins known to be highly expressed in breast cancer cells and tissues, such as NF-κB, β-catenin, cyclin D1, Bcl-2 and survivin. Akt/NF-κB signaling pathway plays a major role in breast carcinogenesis. NF-κB up-regulation is implicated not only in tumor growth and progression, but also in the resistance to chemo- and radiotherapies. Several studies have documented the elevated activity of this protein in breast cancer cells [38,39], which makes it an excellent target for cancer therapy [40,41]. In a recent study, it has been shown that eugenol can inhibit cell proliferation via NF-κB suppression in a rat model of gastric carcinogenesis [42]. The other important breast cancer signaling pathway is the Wnt/β-catenin, which is another transcription factor that has been found highly expressed in various types of cancer, including breast carcinomas [43,44], and is particularly activated in triple negative breast cancer. Therefore, the Wnt/β-catenin signaling pathway constitutes an important potential therapeutic target in the treatment of breast cancer, especially the triple negative form of the disease [45].

The activation of these 2 signaling pathways leads to the up-regulation of cyclin D1, which is a common downstream effector protein. Cyclin D1 is an oncogene that is over-expressed in about 50% of all breast cancer cases [46], and its down-regulation is an important target in breast cancer therapy [47]. Therefore, eugenol-related down-regulation of NF-κB and β-catenin and their common downstream target cyclin D1 could have a great inhibitory effect on breast cancer growth. Importantly*,* the inhibitory effect of eugenol on these onco-proteins was also observed *in vivo* in tumor xenografts (Figure 6).

## Conclusions

Eugenol could constitute a potent anti-breast cancer agent with less side effects than the classical chemotherapeutic agents, through targeting the E2F1/survivin oncogenic pathway. Therefore, eugenol warrants further investigations for its potential use as chemotherapeutic agent against ER-negative and also p53-defective tumors, which are still of poor prognosis.

## Abbreviations

ATCC: American type culture collection; DMSO: Dimethyl sulfoxide; FBS: Fetal bovine serum; RT-PCR: Reverse transcriptase-polymerase chain reaction; GAPDH: Glyceraldehyde-3-phosphate dehydrogenase; shRNA: Short hairpin RNA.

## Competing interests

The authors declare they have no competing interests.

## Authors’ contributions

IA carried out the majority of the experiments. AR conceived the project. AA conceived the project, supervised research and wrote the manuscript. All authors read and approved the final manuscript.

## Pre-publication history

The pre-publication history for this paper can be accessed here:

http://www.biomedcentral.com/1471-2407/13/600/prepub
